# Going against the Herd: Psychological and Cultural Factors Underlying the ‘Vaccination Confidence Gap’

**DOI:** 10.1371/journal.pone.0132562

**Published:** 2015-09-01

**Authors:** Matthew Browne, Patricia Thomson, Matthew Justus Rockloff, Gordon Pennycook

**Affiliations:** 1 School of Human, Health & Social Sciences, Central Queensland University. University Dr, Branyan, QLD 4670, Australia; 2 Department of Psychology, University of Waterloo. 200 University Avenue West, Waterloo, ON N2L 3G1, Canada; 3 School of Health Sciences, University of Stirling, Stirling FK94LA, United Kingdom; Universidad de Granada, SPAIN

## Abstract

By far the most common strategy used in the attempt to modify negative attitudes toward vaccination is to appeal to evidence-based reasoning. We argue, however, that focusing on science comprehension is inconsistent with one of the key facts of cognitive psychology: Humans are biased information processors and often engage in motivated reasoning. On this basis, we hypothesised that negative attitudes can be explained primarily by factors unrelated to the empirical evidence for vaccination; including some shared attitudes that also attract people to complementary and alternative medicine (CAM). In particular, we tested psychosocial factors associated with CAM endorsement in past research; including aspects of spirituality, intuitive (vs analytic) thinking styles, and the personality trait of openness to experience. These relationships were tested in a cross-sectional, stratified CATI survey (N = 1256, 624 Females). Whilst educational level and thinking style did not predict vaccination rejection, psychosocial factors including: preferring CAM to conventional medicine (OR .49, 95% CI .36–.66), endorsement of spirituality as a source of knowledge (OR .83, 95% CI .71–.96), and openness (OR .86, 95% CI .74–.99), all predicted negative attitudes to vaccination. Furthermore, for 9 of the 12 CAMs surveyed, utilisation in the last 12 months was associated with lower levels of vaccination endorsement. From this we suggest that vaccination scepticism appears to be the outcome of a particular cultural and psychological orientation leading to unwillingness to engage with the scientific evidence. Vaccination compliance might be increased either by building general confidence and understanding of evidence-based medicine, or by appealing to features usually associated with CAM, e.g. ‘strengthening your natural resistance to disease’.

## Introduction

A recent nationally representative immunisation survey suggests that approximately 40% of parents in the United States may delay or refuse vaccinations for their children [1]. Parents in this category are significantly more likely to hold negative attitudes towards vaccination and orthodox health care provision in general. This may be partly due to a lack of understanding of the rationale behind public vaccination schemes [2]. Vaccination acceptance may rely more on a willingness to trust advice received by authority Figs [1]; and more generally, one’s level of trust in the evidence-based medical model [3]. These findings parallel results in the field of reasoning and decision-making: that human reasoning is motivated by social and emotional factors, and sometimes falls prey to inaccurate heuristics and biases [4,5]. From this perspective, an accurate understanding of the evidence around vaccination is partially the outcome of a broader attitudinal and psychological stance, and as such is unlikely to change by appeals to reasoning or evidence alone. The goal of the current work is to investigate these psychological and attitudinal determinants of vaccination endorsement.

Recent qualitative research has suggested a number of broader cultural and attitudinal factors that may contribute to negative vaccination attitudes, including: an alignment with alternative/complementary or holistic health, anti-authoritarian worldviews, conspiracy ideation; and certain political, spiritual, or religious identities [3,6–10]. There is some agreement that specific health beliefs such as distrust of medical professionals, and perceived vaccine-efficacy and safety, do predict vaccination attitudes and behaviour [11–14]. However, relatively little attention has been paid to the impact of more distal psychological and attitudinal characteristics on vaccination attitudes. This has led to calls for more research to determine the psychological and social factors that contribute to the “vaccine confidence gap” [15]. Our premise is that individuals differ in terms of their personal style to understanding and making decisions about the world. We shall next discuss our rationale for expecting that certain traits and general attitudes to be associated with negative vaccination attitudes.

### Complementary and alternative therapies

Endorsement of complementary and alternative medicine (CAM) provides a useful counterpoint to acceptance of vaccination. CAM encompasses a diverse range of practices outside the dominant (evidence-based) medical paradigm. They are popular, despite lacking empirical evidence of efficacy, or endorsement by mainstream medical or scientific authorities. The persuasive appeal of CAMs has been explained convincingly in terms of satisfying social and psychological needs, through an advocacy of such concepts as purity, connection to nature, vitalism, and spirituality [16]. Given the marked contrasting features between CAM and vaccination on these dimensions, it is reasonable to consider whether the same qualities that attract some individuals to CAM also explain their rejection of vaccination. CAM usage does appear to be an indicator of individuals who are less likely to use conventional, evidence-based treatments, including vaccination [17]. Parents with a preference for CAM are more sceptical of the benefit of vaccinations for their children, and more likely to believe misconceptions about vaccination [18,19]. Practitioners of CAM, such as homeopaths, have been found to hold negative attitudes towards vaccination [20–22], and school officials who support CAM have been found to also advise against the use of vaccines [23]. Furthermore, naturopaths, who aim to encourage the body’s “inherent self-healing processes” [24] are more likely to oppose vaccination on the basis of being unnecessary, unnatural, and elitist [24,25].

Given that CAM adherence appears to be contra-indicative of vaccination acceptance, it is worthwhile to consider specific appealing properties of CAM and relate them specifically to vaccination scepticism. The valuing of “naturalness”, a key belief associated with CAM, is a specific form of *affect heuristic* [26], and may be just one of a number of cognitive biases (detailed below) that are determinants of favourable attitudes to CAM as well as negative attitudes about vaccination. Some components of a belief system associated with CAM; including a post-modern worldview, a holistic orientation to health and spirituality, and valuing personal growth and self-expression [27]; have also been associated with negative attitudes towards vaccination [3,7]. From this perspective, vaccination treatments ignore holistic and spiritual aspects of health, are an artificial product of the biomedical industry, and are associated with discomfort, and the ‘contamination’ of a healthy body with suspect viral materials. These observations lead us to propose that the folk perception, and experience of, vaccination treatments (e.g. associations with needles and discomfort) make it particularly prone to rejection on the basis of being “unnatural”, and inconsistent with a CAM-centric world-view of health [27,28].

### Inconsistencies in the negative relationship between CAM and vaccination

Despite the arguments and finding above, the relation between CAM and both vaccination attitudes and their uptake in the general population is not clearly resolved. One study reported that coverage levels for influenza and pneumococcal vaccines was significantly higher among adults who were recent or past CAM users [29]. However, as noted by the study authors, this result might be explained by the fact that respondents in that survey tended to use CAM as a *supplemental* rather than *alternative* health care, and utilisation of supplemental therapies reflected a greater overall concern with maintenance of good health. As a group, CAM users have been reported to receive more, rather than less, conventional preventative care [30,31]. However, these positive relationships between CAM usage and preventative conventional health-care have been observed in sub-populations suffering from a chronic condition. Therefore, the positive association may be due to an active approach to managing a chronic condition, which encompasses both conventional and non-conventional treatments.

### Personality and sociocultural factors

The association of CAM endorsement and vaccination rejection may be due to an underlying worldview of some people; which favours a natural, holistic, and “empowered” approach to personal health. Along with use of CAM, distrust of government and authorities is also associated with vaccine refusal [19], whilst political conservatism is associated with greater acceptance of vaccination [6]. An anti-vaccination stance might therefore also be associated with a tendency to reject the status-quo, in the form of advice from perceived establishment authorities; in the form of scientists, medical professionals, government or pharmaceutical companies [15,26–28]. In extreme cases, this may take the form of conspiracy ideation [32].

Distrust of authority, un-conventional, and anti-authoritarian worldviews are related to personality dimensions, most notably the personality dimension *openness to experience* (‘openness’: *inventive/curious* vs. *consistent/cautious*). Openness is a primary dimension of the ‘Big Five’ Five-factor model [33], the product of some decades of factor-analytic personality research and arguably the standard descriptive model of personality traits. Positive scores on this dimension are conceptually related to the identified “post-modern” culture associated with anti-vaccination movement [3,7]; leading to rejection of purportedly objective knowledge from conventional authority sources, in favour of a personal, emotional, and spiritual approach to health decisions. However, personality factors have not yet been specifically associated with attitudes towards vaccination.

### Cognitive factors

A further psychological rationale for a negative link between CAM and vaccination adherence is based on individual differences in analytic decision-making. The rationale for vaccination involves judgement under uncertainty, as well as appropriate weighting of low-probability events [4,34]. Heuristics that have been argued to affect decision making about vaccination include *compression*: over-estimating the frequency of rare risks, and *availability*: over-estimating the frequency of events that are more accessible or easily remembered [35]. The efficacy of vaccination programs, in virtually eliminating the 1^st-^ or 2^nd^-hand experience of many preventable diseases in developed countries, tends to increase the impact of the availability bias, leading to discounting of the risk of non-vaccination. In contrast, the relatively common occurrence of mild discomfort or stress during childhood immunisations, or mild and transient reactions, tend to be over-weighted due to their relative frequency and immediacy [36]. In addition, o*mission bias* plays a role in vaccination decisions, in which a negative consequence from inaction (not getting a vaccine) is weighted less heavily than a negative outcome from an action (getting the vaccine) [37,38]. Finally, a bias has been shown towards natural risks (i.e., disease) being generally more acceptable than man-made risks (e.g., vaccination reactions) [39].

Increasing attention is being paid to individual differences in the willingness to thinking analytically about problems, which is counter-indicative of susceptibility to biases in judgement and decision making [40]. The Cognitive Reflection Test [41] (CRT), in particular, has been shown to have high predictive efficacy; predicting the ability to choose options with higher expected value and resistance to the logical fallacies described above [42,43]. Such measures fall within dual-process theories of human cognition, which distinguish between “intuitive” reasoning; which is fast, automatic and does not require working memory; and “analytical” reasoning, which is slow, deliberative, and requires working memory [44]. An analytical mode of reasoning, associated with more sound empirical and causal judgements, might be expected to yield a more favourable evaluation of vaccination in line with available evidence. However, no research has specifically considered individual differences in cognitive style and reasoning as factors predicting attitudes towards vaccination.

### Aims

Previous research is highly suggestive regarding the impact of psychosocial factors that may determine formation of attitudes towards vaccination. The current study is based on the supposition that negative attitudes towards vaccination may be influenced primarily by cultural and psychosocial factors, rather than by evidence-based analytic reasoning. We aimed to test this idea, and gauge the relative impact of these factors through bivariate and multivariate associations in a cross-sectional design. Specifically, the present study aimed to assess the degree to which rejection of vaccination is associated with four cognitive and social factors:

1. Lowered willingness to consider the empirical evidence (indicated by an intuitive rather than analytical cognitive style)
2. Unwillingness to trust information delivered by conventional authority sources or to conform to social norms (indicated by openness to experience)
3. Adherence to CAM-centric health values (indicated by CAM utilisation and preference for CAM over conventional treatment)
4. A non-evidence-based world-view (indicated by a spiritual approach to knowledge)

We expect that all four individual characteristics should be positively associated with vaccination rejection. However, we are specifically interested in the relative contribution of cognitive style (1) versus openness to experience (2). This sheds light on the question of whether vaccination rejection reflects an unwillingness to effectively process the empirical evidence in support of vaccines; or alternatively (1), or reflect a psychosocial mindset that rejects engaging in a fair cognitive evaluation (e.g., due to lack of faith in the scientific method, or distrust of conventional authoritative sources of information). Finally, we note that the four determinants listed would be expected to be positively associated with one another. Nevertheless, multivariate analysis provides scope for determining the relative contribution of each factor.

## Materials and Methods

A cross-sectional survey was used to obtain data on attitudes to vaccination along with other attitudinal, cognitive and personality variables. Data was collected in the Australian state of Queensland, as part of the 2012 Queensland Social Survey (QSS)[45]. A two-stage stratified sampling strategy was used to select both households and individuals randomly in Queensland. The sample was derived from the commercially available Electronic White Pages using a computer program to select a simple random sample of domestic phone numbers. Within each contacted household, one eligible person was selected at random using the first birthday method. Potential interviewees were screened based on age (18 years or older), and preferentially selected by sex (male or female) to meet an even quota of male and female respondents for the whole survey. Sampling with respect to geographical (e.g. metropolitan versus rural areas) and demographic (e.g. gender) was tailored to be representative of the general population of Queensland; full details of which are provided in the sampling report [45]. However, compared to national census data [46], there was an over-sampling in the over-55 category, and under-sampling in the under-35 age category. Verbal consent (due to the telephone interview format) was obtained from all participants before commencement. The protocol included a mandatory screen that the interviewer must read to the participant; requiring selection of checkbox indicating participant consent before proceeding. Prior ethical approval for the study was received from the Human Ethics Research Review Panel at Central Queensland University (Project: H10/06-121, QSS 2012). Ethical approval included approval of the verbal consent procedure used in this study. Due to restrictions on survey length, validated short-form or partial versions of self-report instruments were generally preferred, as detailed in the following sections. The dataset analysed is provided as supporting information to this article (**S1 Dataset**).

Respondents provided a variety of demographic variables that were potentially related to vaccination endorsement. Marital status was recoded to a simple binary measure, with de facto relationships and formally married individuals combined and contrasted with those not currently in a relationship: single, widowed, and divorced individuals. For the purposes of summarizing baseline prevalence and bivariate associations, binary contrasts were also constructed for age (49+), household income (>median: $43,100), number of children (1+), and region (metropolitan versus rural or regional).

### Complementary and alternative therapies

Questions regarding CAM were derived from a previously validated instrument, designed to standardize measurement of CAM utilization[47], and distinguish between those that use a particular CAM from those that do not [48,49]. Each CAM item provided an indication of whether the respondent had utilized each of the following therapeutic or self-treatment activities within the last 12 months: herbal and homeopathic remedies, energy-based and body therapies (including therapeutic massage), vitamins, yoga, meditation, prayer, body therapies, hypnosis, spiritual healing, and chiropractic or osteopathic treatments.

### Personal beliefs and attitudes

The items described above were intended to quantify CAM utilization, rather than a preference for CAM over conventional medicine. Two further items were included, the first intended to measure a preference for CAM over conventional medical treatments [50]; “If I was suffering from an illness or injury I would try an alternative therapy before seeking conventional medical treatment”. The second was taken from the internal health locus of control scale, and designed to indicate perceived control over health [51]; “If I get sick, it is my own behaviour that determines how soon I get well again”.

Indicators of general religious and spiritual beliefs were also included. Participants indicated their religious denomination by selecting from a list given by the interviewer based on Australian census data. Participants could also indicate their denomination if it was not on the list given. Slightly over 1/3 of respondents (n = 391, 34.9%) selected “no religious affiliation (agnostic / atheist)” as their religious affiliation. Over half of respondents (n = 702, 63.2%) indicated either Catholic, or a mainstream Protestant denomination, with the remainder including a diverse range of affiliations; including, Jehovah’s Witness, Church of Latter Day Saints (Mormon), Seventh Day Adventist, and Assembly of God. Participants were also asked the yes/no question “Would you say your religious beliefs are very strong?” (hereafter “strong faith” for analysis). A final item, designed to indicate a spiritual or revelatory epistemology [52,53] rather than religious belief, was included; “I believe that most important knowledge comes from spiritual experiences”, scored on a 7-point Likert scale. This item was intended to capture that aspect of religiosity / spirituality that was antithetical to an evidence-based world-view. Thus, three indicators of faith and spirituality were considered: possessing a denominational affiliation, strength of religious faith, and spiritual epistemology. Some more conservative religious faiths are known to proscribe vaccination and other modern health practices. However, as these are of very low prevalence in Australia, we expected only spiritual epistemology to be related to vaccination attitudes.

### Personality / cognitive style

We included a brief 3-item measure of the Openness dimension of the global Five-factor personality model, which has demonstrated good external and convergent validity, as well test-retest reliability [54]. Participants were asked to indicate to what extent they agreed with three statements: “I am open to new experiences”, “I am a creative person”, and “I am a complex and unconventional person”. Responses were made on a 7-point Likert scale (1 = “Strongly disagree”, 2 = “Disagree moderately”, 3 = “Disagree a little”, 4 = “Neither agree or disagree”, 5 = “Agree a little”, 6 = “Agree moderately”, 7 = “Strongly agree”). Our observed alpha reliability was low (.42), similar to that reported by the developers (.45) [54], who demonstrate that this apparent deficiency is due to the bias of alpha when calculated for very short scales. Also included was the 3-item Cognitive Reflection Test (CRT) [41], that indexes participants’ tendency to override an initial intuitive response by applying analytic thinking skills. For example, the first item is: “If it takes 5 machines 5 minutes to make 5 widgets, how long would it take 100 machines to make 100 widgets?” The intuitive answer for this question is 100, whereas the correct answer is 5. The CRT may also be understood as measuring an individual’s inclination towards miserly information processing, and is the most popular instrument based on modern dual process theories of cognition; itself a major theme during the last 50 years of research in cognitive science [55]. The CRT has proven to be a potent predictor of performance on various rational thinking tasks [55] including, a tendency to choose high expected-value gambles, temporal discounting, maximising strategies on probabilistic prediction tasks, and non-superstitious thinking [40,56,57].

### Vaccination scepticism / endorsement

Australia has a comprehensive public vaccination program [58], with eligibility for certain government benefits dependent on children receiving all scheduled vaccinations. Vaccination items were drawn from a previous study [12], with the insertion of ‘recommended’ or ‘scheduled’ before immunization, to reflect the Australian context. We aimed to capture both attitudes regarding safety and behavioural intentions. Perceived safety and intended behaviour were assessed by two items, “If I had a baby to care for, I would want him/her to get all the recommended immunizations”, and “I believe that scheduled immunizations are safe for children”, with responses on the 7 point Likert scale described above added to create a composite score with a maximum of 14; lower scores indicating greater vaccination scepticism. Standardized coefficient alpha for the aggregate measure was 0.85.

## Results

Vaccination endorsement was heavily skewed towards the positive, with 887 (70.6%) respondents indicating strong agreement (scoring 14), 194 (15.4%) agreeing moderately (scoring 12–13), 61 agreeing a little (scoring 10–11), and 114 (9.1%) neutral or negative in their attitudes (scoring <= 9). Accordingly this variable was recoded in these four categories for subsequent analyses. Table 1 provides descriptive statistics and bivariate associations between vaccination endorsement and all other variables, each treated as a single contrast, except for occupational category, which had three levels. For example, the first row of Table 1 shows that overall 888 out of 1256, or 70.7%, of respondents were currently in a married or de facto relationship. Of those with lowest level of vaccination endorsement, 59.6% were married, whilst in the group with the highest vaccination endorsement, 73.4% were married. Note that the categories for CRT and Openness scores were defined arbitrarily for the purpose of conveniently describing these relationships in this table. These and other covariates are treated as per their original continuous scoring in subsequent analysis.

**Table 1:** Bivariate association with four levels of vaccination endorsement: demographics, CAM utilization and personal characteristics of the respondent.

				Vaccination endorsement									
				Neutral/ Negative		Little		Moderate		Strong		*χ* ^2^	*p*
		Base prevalence		9.1%	(114)	4.8%	(61)	15.4%	(194)	70.6%	(887)		
*Demographics*													
	Married	70.7%	(888/1256)	59.6%	(68)	70.5%	(43)	64.9%	(126)	**73.4%**	**(651)**	**12.9**	**0.006**
	Aged 49+	64.2%	(799/1244)	66.1%	(74)	48.3%	(29)	60.6%	(117)	65.9%	(579)	8.9	0.033
	1+ children	35.4%	(444/1256)	32.5%	(37)	47.5%	(29)	32.5%	(63)	35.5%	(315)	5.1	0.163
	Language other than English	7.6%	(95/1252)	12.3%	(14)	4.9%	(3)	7.3%	(14)	7.2%	(64)	4.4	0.229
	Gender (female)	49.7%	(624/1256)	46.5%	(53)	60.7%	(37)	51.0%	(99)	49.0%	(435)	3.7	0.299
	Region (metropolitan)	60.1%	(753/1252)	57.9%	(66)	63.9%	(39)	59.1%	(114)	60.4%	(534)	0.7	0.863
	*Occupational category*													
	White collar		(117/717)	Ref.		Ref.		Ref.		Ref.				
	Blue collar	19.9%	(143/717)	15.6%	(10)	15.4%	(6)	20.9%	(24)	20.6%	(103)	1.5	0.712
	Professional	63.7%	(457/717)	60.9%	(39)	61.5%	(24)	59.1%	(68)	65.3%	(326)	1.9	0.595
	Household income > median	50.8%	(402/792)	47.2%	(34)	57.1%	(24)	47.3%	(61)	51.5%	(283)	1.8	0.605
*Alternative therapies used last 12 months*														
	Herbalist	29.1%	(366/1256)	**42.1%**	**(48)**	**45.9%**	**(28)**	**36.6%**	**(71)**	24.7%	(219)	**31.3**	**<0.001**
	Energy-based	13.4%	(168/1256)	**21.1%**	**(24)**	**29.5%**	**(18)**	11.3%	(22)	11.7%	(104)	**22.3**	**<0.001**
	Homeopathic	10.7%	(134/1256)	**20.2%**	**(23)**	**19.7%**	**(12)**	**12.9%**	**(25)**	8.3%	(74)	**22**	**<0.001**
	Vitamins	61.9%	(778/1256)	**75.4%**	**(86)**	**67.2%**	**(41)**	**69.6%**	**(135)**	58.2%	(516)	**19.7**	**<0.001**
	Yoga	9.3%	(117/1256)	**16.7%**	**(19)**	**14.8%**	(9)	**12.9%**	**(25)**	7.2%	(64)	**17**	**<0.001**
	Meditation	14.4%	(181/1256)	**25.4%**	**(29)**	**19.7%**	**(12)**	**15.5%**	**(30)**	12.4%	(110)	**15.7**	**0.001**
	Other	2.9%	(36/1256)	**7.0%**	**(8)**	**6.6%**	**(4)**	2.1%	(4)	2.3%	(20)	**11.7**	**0.009**
	Prayer	13.4%	(168/1256)	**20.2%**	**(23)**	**19.7%**	**(12)**	**14.9%**	**(29)**	11.7%	(104)	**9.1**	**0.028**
	Body therapies	31.4%	(395/1256)	**36.1%**	**(41)**	**42.6%**	**(26)**	**36.0%**	**(70)**	29.1%	(258)	**8.8**	**0.031**
	Hypnosis	1.6%	(20/1256)	2.6%	(3)	4.9%	(3)	0.5%	(1)	1.5%	(13)	6.6	0.085
	Spiritual	3.4%	(43/1256)	7.0%	(8)	4.9%	(3)	3.6%	(7)	2.8%	(25)	5.9	0.118
	Chiropractic/osteopathy	31.8%	(400/1256)	36.0%	(41)	39.3%	(24)	35.1%	(68)	30.1%	(267)	4.6	0.201
*Beliefs / attitudes*														
	Prefer CAM over conventional therapy	25.3%	(316/1247)	**45.5%**	**(51)**	**42.6%**	**(26)**	**29.2%**	**(56)**	20.7%	(183)	**45.1**	**<0.001**
	Knowledge from spiritual experiences	44.2%	(546/1235)	**60.4%**	**(67)**	**48.3%**	**(29)**	**38.7%**	**(74)**	43.1%	(376)	**14.9**	**0.003**
	Strong faith	48.8%	(413/847)	55.7%	(39)	50.0%	(20)	43.8%	(60)	49.0%	(294)	2.7	0.437
	No religious denomination	31.7%	(391/1234)	38.4%	(43)	31.7%	(19)	30.4%	(58)	31.1%	(271)	2.6	0.469
	Health locus of control	68.4%	(835/1221)	64.0%	(71)	72.9%	(43)	70.2%	(134)	68.3%	(587)	1.8	0.616
*Personality / cognitive style*														
	CRT score > 0	49.8%	(626/1256)	53.5%	(61)	47.5%	(29)	44.3%	(86)	50.7%	(450)	3.4	0.336
	CRT score > 1	14.8%	(186/1256)	11.4%	(13)	4.9%	(3)	16.0%	(31)	15.7%	(139)	6.5	0.085
	Openness score > 9	74.5%	(930/1248)	82.5%	(94)	81.7%	(49)	73.6%	(142)	73.2%	(645)	6.3	0.101
	Openness score > 12	31.7%	(395/1248)	**40.3%**	(46)	**38.3%**	(23)	24.8%	(48)	31.3%	(278)	9.3	**0.025**

Note: *p*-values < .05 shown in bold. For these indicators, the levels of vaccination endorsement with higher than baseline % responses are also shown in bold.

With the exception of marital status, no demographic variables were found to discriminate attitudes towards vaccination. However, those aged 49+ tended to have more unambiguous views (i.e. at either extreme of vaccination attitudes; *p* = .033). Health characteristics and health behaviours also did not individually significantly discriminate different levels of vaccination endorsement, nor did cognitive or personality factors. However, effects for both CRT>1 (*p* = .085) and Openness>12 (*p* = .025) were in the expected direction. Of the belief and attitudinal variables, religious characteristics and health locus of control were not significant. However, indicating a spiritual epistemology was quite clearly associated with lower levels of vaccination endorsement (*p* = .003), as was a preference for CAM over conventional medical treatments (*p* < .001). Utilization of most individual alternative therapies in the last 12 months was associated with increased vaccination scepticism. Additionally, the rank-order correlation between the number of different alternative therapies used in the last 12 months and vaccination attitude score was significant, *p* = −.18, *p* < .0001.

Multivariate tests of association were done using ordinal logistic regression, summarised in Table 2. All models include basic demographic covariates, Openness, and CRT as predictors. Models were considered with and without the significant attitudinal (preferring CAM, and spiritual epistemology) variables. Considering the sampling bias towards older respondents and the relevance of parenting age for vaccination decisions, the analyses were repeated for the subset (N = 406) of participants’ aged <50. The odds ratios shown for the ordinal response models are interpreted in much the same way as a binary model, describing the odds of a higher category of response for a one-unit increase in the predictor. Openness to experience, but not analytical thinking, was generally associated with more negative attitudes to vaccination (*p* < .01) when demographic covariates were controlled for. The effect size for Openness decreased with the inclusion of the CAM / attitudinal variables, suggesting shared explanatory covariance. This is congruent with observed positive covariation in the present sample between Openness and preferring CAM over conventional treatment, Spearman’s rank-order correlation *ρ* = .11, *p* = .0001; and Openness and a spiritual epistemology, *ρ* = .20, *p* < .0001. The pattern of effects was generally similar for the analyses of the whole sample compared with younger subset, with certain effects non-significant due to the smaller sample size.

**Table 2:** Ordinal logistic models predicting endorsement of vaccination from demographic, psychological and attitudinal characteristics.

			All participants					* *	Aged <50						
			(1)		(2)		(3)			(1)		(2)		(3)	
		M[SD] or N	OR [95% CI]	*p*	OR [95% CI]	*p*	OR [95% CI]	*p*	M[SD] or N	OR [95% CI]	*p*	OR [95% CI]	*p*	OR [95% CI]	*p*
*Demographic*															
	Age	55.2[15.6]	**1.20 [1.04; 1.37]**	**.*01***	**1.20 [1.04; 1.37]**	**.*01***	1.13 [0.98; 1.30]	.*09*	38.3[8.6]	1.31 [0.90; 1.91]	.*15*	1.31 [0.90; 1.91]	.*16*	1.24 [0.84; 1.82]	.*27*
	Gender (Female)	N = 624	0.78 [0.59; 1.02]	.*07*	0.84 [0.63; 1.11]	.*21*	0.88 [0.66; 1.17]	.*36*	N = 219	0.68 [0.44; 1.06]	.*08*	0.72 [0.46; 1.12]	.*15*	0.76 [0.48; 1.19]	.*22*
	Education^a^ (Years)	13.8[4.1]	1.06 [0.91; 1.23]	.*46*	1.06 [0.92; 1.23]	.*44*	1.03 [0.89; 1.19]	.*73*		0.92 [0.72; 1.20]	.*54*	0.92 [0.71; 1.19]	.*53*	0.88 [0.68; 1.14]	.*33*
	No Religion		1.04 [0.78; 1.40]	.*78*	0.93 [0.69; 1.27]	.*66*	0.97 [0.72; 1.32]	.*86*		1.00 [0.65; 1.56]	.*99*	0.92 [0.59; 1.45]	.*71*	0.95 [0.60; 1.50]	.*82*
*Personality / cognition*															
	CRT	.45[.76]	0.91 [0.77; 1.09]	.*32*	0.90 [0.75; 1.07]	.*22*	0.89 [0.75; 1.07]	.*20*	.54[.82]	0.92 [0.71; 1.20]	.*53*	0.90 [0.70; 1.18]	.*45*	0.91 [0.70; 1.19]	.*47*
	Openness^a^	4.9[1.2]	**0.79 [0.68; 0.91]**	***<* .*01***	**0.83 [0.71; 0.96]**	**.*01***	**0.86 [0.74; 0.99]**	**.*03***	**5.1[1.1]**	**0.69 [0.54; 0.87]**	***<* .*01***	**0.72 [0.56; 0.92]**	***<* .*01***	**0.76 [0.59; 0.97]**	**.*03***
*Attitudinal*															
	Spiritual^a^	3.2[2.1]			**0.80 [0.69; 0.92]**	***<* .*01***	**0.83 [0.71; 0.96]**	**.*01***	3.3[2.0]			0.84 [0.66; 1.07]	.*159*	0.88 [0.69; 1.12]	.*28*
	Prefer CAM	N = 316					**0.49 [0.36; 0.66]**	***<* .*01***	N = 129					**0.42 [0.27; 0.66]**	***<* .*01***
*Intercepts* ^b^															
			0.06|0.10|0.30		0.05|0.10|0.29		0.04|0.09|0.25			0.04|0.09|0.23		0.04|0.08|0.24		0.03|0.07|0.19	
	AIC		1810.5		1803.0		1783.5			756.3		756.3		744.05	* *
	Num. obs.		1085		1085		1085			406		406		406	

Note: *p*-values < .05 shown in bold.

^a^ normalized for purpose of calculating odds ratio

^b^ CI for intercepts not shown, base odds given for category thresholds: neutral/negative *vs* agree a little | agree a little *vs* agree moderately | agree moderately *vs* agree strongly.

Higher CRT scores (indicating a more analytical thinking style) were negatively related to a preference for CAM over conventional medical treatment *ρ* = −.12, *p* < .0001, as well as the number of CAM treatments used in the last year *ρ* = −.08, *p* = .0047. Finally, analytical thinking style was negatively related to all forms of CAM, with this relationship significant in three cases; herbal remedies *ρ* = −.08, *p* = .0014, homeopathy, *ρ* = −.06, *p* = .0236, and prayer for the purpose of healing, *ρ* = −.15, *p* < .0001.

## Discussion

Utilisation of a broad range of CAM, and a preference for CAM over conventional treatments, was found to be the strongest predictor of negative vaccination attitudes. The CAMs most strongly linked to vaccination rejection were those with a spiritual or philosophical basis (e.g. homeopathy) rather than those more closely aligned to the conventional medical paradigm (e.g. chiropractic). Additional effects were found for the personality construct of openness to experience, and a spiritual orientation to knowledge. Multivariate analyses confirmed that these three characteristics contribute unique explanatory power in vaccination scepticism. However, the effect sizes of the two psychosocial factors decreased slightly with inclusion of the CAM variable, suggesting shared explanatory effects. A significant effect for analytic versus intuitive cognitive style was not found; suggesting that it is a cultural and personal orientation, rather than cognitive ability, that has a direct influence on vaccination attitudes.

Taken together, these results describe a vaccine sceptic as viewing themselves as anti-authoritarian and unconventional, with a preference for unorthodox treatments with spiritual or ‘life-affirming’ features. The significant effect for personality, but not for cognitive style, is congruent with the notion that it is a reluctance to engage with the evidence, rather than a lack of capacity to appropriately process the evidence, that predicts vaccination scepticism. However, this interpretation requires further investigation before it can be held with high confidence.

Of most practical relevance is the finding that anti-vaccination attitudes are related to a broader personality and attitudinal mindset encompassing spirituality, openness to experience, and CAM adherence. One attractive interpretation of this covariation is that vaccination attitudes are determined by whether one subscribes to the conventional, authoritative, and evidence-based medical model. This suggests that further presentation of objective evidence, specific to vaccination, may not address the ‘vaccine confidence gap’ [15]. A more effective strategy may be to build *general* confidence in a scientific, evidence-based approach to health. Also, vaccination might be promoted in such a way as to appeal to the socio-emotional criteria that some individuals use to make health decisions.

As expected, higher scores on the openness personality dimension were negatively related to vaccination endorsement; and as shown in Table 2, some of the shared covariance is shared with spiritual thinking and preference for CAM over conventional treatments. Therefore, given that openness was strongly associated with preferring CAM, these data supports the interpretation that the same psychosocial factors, namely openness to alternatives, may lead to an attraction towards CAM and rejection of vaccination. Similarly, spirituality, but not conventional indicators of religiosity, was associated with vaccination rejection. A spiritual orientation to knowledge and decision-making is arguably a key characteristic of many CAMs, and is somewhat antithetical to the evidence-based approach that underlies vaccination. Conventional religiosity, on the other hand, tends to be associated with social conservatism, and acceptance of advice from trusted authority Figs, which is consistent with the results and theoretical perspectives presented here.

Contrary to expectations, cognitive style did not predict vaccination endorsement. Although the atypical telephone interview method of administration in this study suggests caution in interpreting this null result, the likewise failure of education to predict vaccination endorsement converges on a similar conclusion: analytic thinking plays little to no *direct* role in determining attitudes toward vaccination. While there is evidence for an *indirect* role as CRT performance successfully predicted scepticism about CAMs and an analytic cognitive style has been associated with spiritual [59], religious [60], paranormal [56], and conspiratorial [61] thinking, the lack of direct association nonetheless suggests that negative attitudes toward vaccination are a consequence of cultural (as opposed to primarily analytic cognitive) forces. At the very least, these results call into question the potential efficacy of fact-based strategies. A misunderstanding of failure to entertain the facts and evidence regarding vaccination do not appear to be the source of negative attitudes.

### Limitations

As with all cross-sectional studies, the results are limited to measures of association and can only be indicative of potential causal paths of attitude formation. The breadth of variables measured led to restrictions on the number of items available to measure individual constructs. The brief form of the openness to experience scale would be better administered through in a much longer, multidimensional format. Whilst the CRT measure of cognitive style was used in its original form, it has not previously been used in a phone-interview format. The relatively poor performance of the sample compared to norms may evidence diminished reliability on this measure. Finally, it is regrettable that a standard recognised scale for measuring vaccination acceptance / scepticism is not yet available. Our measure of vaccination attitudes was limited in terms of the number of items used, and should be taken to provide only a indication of general attitudes towards vaccination. In social psychology, the compatibility principle encapsulates the common observation that general attitudes are poor predictors of specific attitudes or behaviours [62]. This implies caution in linking general vaccination attitudes to, for example, a decision to accept a MMR vaccine. Psychometric development and validation of a standard vaccination scepticism / endorsement scale is highly recommended.

## Conclusion

CAM endorsement and vaccination scepticism are components of a common attitudinal stance, with some shared psychosocial determinants. The results of the present study indicate that vaccination rejection is related to psychosocial factors: a general preference for complementary over conventional medicines, valuing diverse and unconventional alternatives, and a spiritual orientation to attitude formation. The null findings with regard to cognitive style and educational level suggest that factors unrelated to the actual empirical evidence for vaccination – i.e. a particular personality and attitudinal mindset are most instrumental in determining vaccination attitudes. Efforts to counter vaccination concerns should be mindful that negative vaccination views appear to form part of a broader attitudinal system that does not necessarily trust empirical or positivist evidence from authoritative sources. Vaccination promotion efforts may benefit from targeting groups associated with CAM and building general confidence in scientific medicine, rather than targeting specific misunderstandings regarding vaccination.

## Supporting Information

S1 Dataset Complete dataset analysed for the current study in CSV format. Variables are included in the same order as they appear in Table 1, and coded as per the reported analyses.
(CSV)

## References

[1] Smith PJ, Humiston SG, Marcuse EK, Zhao Z, Dorell CG, Howes C, et al Parental Delay or Refusal of Vaccine Doses, Childhood Vaccination Coverage at 24 Months of Age, and the Health Belief Model. Public Health Rep. 2011;126: 135–146. 21812176 10.1177/00333549111260S215 PMC3113438

[2] Downs JS, de Bruin WB, Fischhoff B. Parents’ vaccination comprehension and decisions. Vaccine. 2008;26: 1595–1607. 10.1016/j.vaccine.2008.01.011 18295940

[3] Kata A (2010). A postmodern Pandora’s box: Anti-vaccination misinformation on the Internet. Vaccine. 2010;28: 1709–1716. 10.1016/j.vaccine.2009.12.022 20045099

[4] Tversky A, Kahneman D. Judgment under Uncertainty: Heuristics and Biases. Science. 1974;185: 1124–1131. 10.1126/science.185.4157.1124 17835457

[5] Stanovich KE, West RF. Individual differences in reasoning: implications for the rationality debate? Behav Brain Sci. 2000;23: 645–665. 11301544 10.1017/s0140525x00003435

[6] Lewandowsky S, Gignac GE, Oberauer K. The Role of Conspiracist Ideation and Worldviews in Predicting Rejection of Science. PLoS ONE. 2013;8: e75637 10.1371/journal.pone.0075637 24098391 PMC3788812

[7] Kata A. Anti-vaccine activists, Web 2.0, and the postmodern paradigm–An overview of tactics and tropes used online by the anti-vaccination movement. Vaccine. 2012;30: 3778–3789. 10.1016/j.vaccine.2011.11.112 22172504

[8] Briones R, Nan X, Madden K, Waks L. When Vaccines Go Viral: An Analysis of HPV Vaccine Coverage on YouTube. Health Commun. 2011;27: 478–485. 10.1080/10410236.2011.610258 22029723

[9] Bean SJ. Emerging and continuing trends in vaccine opposition website content. Vaccine. 2011;29: 1874–1880. 10.1016/j.vaccine.2011.01.003 21238571

[10] Yaqub O, Castle-Clarke S, Sevdalis N, Chataway J. Attitudes to vaccination: A critical review. Soc Sci Med. 2014;112: 1–11. 10.1016/j.socscimed.2014.04.018 24788111

[11] Prislin R, Dyer J. A., Blakely C. H., & Johnson C. D. Immunization status and sociodemographic characteristics: the mediating role of beliefs, attitudes, and perceived control. Am J Public Health. 1998;88: 1821–1826. 9842380 10.2105/ajph.88.12.1821 PMC1509037

[12] Gust DA, Strine TW, Maurice E, Smith P, Yusuf H, Wilkinson M, et al Underimmunization among children: effects of vaccine safety concerns on immunization status. Pediatrics. 2004;114: e16–22. 15231968 10.1542/peds.114.1.e16

[13] Furnham A. Are modern health worries, personality and attitudes to science associated with the use of complementary and alternative medicine? Br J Health Psychol. 2007;12: 229–243. 10.1348/135910706X100593 17456283

[14] Poethko-Müller C, Ellert U, Kuhnert R, Neuhauser H, Schlaud M, Schenk L. Vaccination coverage against measles in German-born and foreign-born children and identification of unvaccinated subgroups in Germany. Vaccine. 2009;27: 2563–2569. 10.1016/j.vaccine.2009.02.009 19428862

[15] Larson HJ, Cooper LZ, Eskola J, Katz SL, Ratzan S. Addressing the vaccine confidence gap. The Lancet. 2011;378: 526–535. 10.1016/S0140-6736(11)60678-8 21664679

[16] Kaptchuk TJ, Eisenberg DM. The Persuasive Appeal of Alternative Medicine. Ann Intern Med. 1998;129: 1061–1065. 10.7326/0003-4819-129-12-199812150-00011 9867762

[17] Jones L, Sciamanna C, Lehman E. Are those who use specific complementary and alternative medicine therapies less likely to be immunized? Prev Med. 2010;50: 148–154. 10.1016/j.ypmed.2009.12.001 20005248

[18] Gellin BG, Maibach EW, Marcuse EK. Do parents understand immunizations? A national telephone survey. Pediatrics. 2000;106: 1097–1102. 11061781 10.1542/peds.106.5.1097

[19] Salmon DA, Moulton LH, Omer SB, DeHart MP, Stokley S, Halsey NA. Factors associated with refusal of childhood vaccines among parents of school-aged children: a case-control study. Arch Pediatr Adolesc Med. 2005;159: 470–476. 10.1001/archpedi.159.5.470 15867122

[20] Ernst E. The attitude against immunisation within some branches of complementary medicine. Eur J Pediatr. 1997;156: 513–515. 9243229 10.1007/s004310050650

[21] Ernst E. Rise in popularity of complementary and alternative medicine: reasons and consequences for vaccination. Vaccine. 2001;20: 90–93. 10.1016/S0264-410X(01)00290-0 11587822

[22] Lehrke P, Nuebling M, Hofmann F, Stoessel U. Attitudes of homoeopathic physicians towards vaccination. Vaccine. 2001;19: 4859–4864. 10.1016/S0264-410X(01)00180-3 11535339

[23] Salmon DA, Moulton LH, Omer SB, Chace LM, Klassen A, Talebian P, et al Knowledge, Attitudes, and Beliefs of School Nurses and Personnel and Associations With Nonmedical Immunization Exemptions. Pediatrics. 2004;113: e552–e559. 15173536 10.1542/peds.113.6.e552

[24] Wilson K, Mills E, Boon H, Tomlinson G, Ritvo P. A survey of attitudes towards paediatric vaccinations amongst Canadian naturopathic students. Vaccine. 2004;22: 329–334. 10.1016/j.vaccine.2003.08.014 14670313

[25] Halper J, Berger LR. Naturopaths and Childhood Immunizations: Heterodoxy Among the Unorthodox. Pediatrics. 1981;68: 407–410. 7279469

[26] Carlisle E, Shafir E. Heuristics and biases in attitudes towards herbal medicines. The shape of reason: essays in honour of Paolo Legrenzi. 2005 pp. 205–223.

[27] Astin JA. Why patients use alternative medicine: Results of a national study. JAMA. 1998;279: 1548–1553. 10.1001/jama.279.19.1548 9605899

[28] Beyerstein BL. Alternative medicine and common errors of reasoning. Acad Med J Assoc Am Med Coll. 2001;76: 230–237. 10.1097/00001888-200103000-00009 11242572

[29] Stokley S, Cullen KA, Kennedy A, Bardenheier BH. Adult vaccination coverage levels among users of complementary/alternative medicine–results from the 2002 National Health Interview Survey (NHIS). BMC Complement Altern Med. 2008;8: 6 10.1186/1472-6882-8-6 18294382 PMC2266896

[30] Egede LE, Ye X, Zheng D, Silverstein MD. The Prevalence and Pattern of Complementary and Alternative Medicine Use in Individuals With Diabetes. Diabetes Care. 2002;25: 324–329. 10.2337/diacare.25.2.324 11815504

[31] Garrow D, Egede LE. Association Between Complementary and Alternative Medicine Use, Preventive Care Practices, and Use of Conventional Medical Services Among Adults With Diabetes. Diabetes Care. 2006;29: 15–19. 10.2337/diacare.29.01.06.dc05-1448 16373889

[32] Jolley D, Douglas KM. The Effects of Anti-Vaccine Conspiracy Theories on Vaccination Intentions. PLoS ONE. 2014;9: e89177 10.1371/journal.pone.0089177 24586574 PMC3930676

[33] Piedmont RL. Five Factor Model of Personality. Encycl Qual Life Well- Res. 2014; 2282–2282.

[34] Kahneman D, Tversky A. Prospect Theory: An Analysis of Decision under Risk. Econometrica. 1979;47: 263 10.2307/1914185

[35] Ball LK, Evans G, Bostrom A. Risky business: Challenges in vaccine risk communication. Pediatrics. 1998;101: 453 9481013 10.1542/peds.101.3.453

[36] Bennett P, Smith C. Parents attitudinal and social influences on childhood vaccination. Health Educ Res. 1992;7: 341–348. 10.1093/her/7.3.341 10148742

[37] Ritov I, Baron J. Reluctance to vaccinate: Omission bias and ambiguity. J Behav Decis Mak. 1990;3: 263–277. 10.1002/bdm.3960030404

[38] Asch DA, Baron J, Hershey JC, Kunreuther H, Meszaros J, Ritov I, et al Omission Bias and Pertussis Vaccination. Med Decis Making. 1994;14: 118–123. 10.1177/0272989X9401400204 8028464

[39] Covello VT, Sandman P, Slovic P. Guidelines for communicating information about chemical risks effectively and responsibly. Accept Evid Sci Values Risk Manag. 1991; 66–90.

[40] Liberali JM, Reyna VF, Furlan S, Stein LM, Pardo ST. Individual Differences in Numeracy and Cognitive Reflection, with Implications for Biases and Fallacies in Probability Judgment. J Behav Decis Mak. 2012;25: 361–381. 10.1002/bdm.752 23878413 PMC3716015

[41] Frederick S. Cognitive Reflection and Decision Making. J Econ Perspect. 2005;19: 25–42. 10.2307/4134953

[42] Campitelli G, Labollita M. Correlations of cognitive reflection with judgments and choices. Judgm Decis Mak. 2010;5: 182–191.

[43] Cokely ET, Kelley CM. Cognitive abilities and superior decision making under risk: A protocol analysis and process model evaluation. Judgm Decis Mak. 2009;4: 20–33.

[44] Evans JSBT. Dual-processing accounts of reasoning, judgment, and social cognition. Annu Rev Psychol. 2008;59: 255–278. 10.1146/annurev.psych.59.103006.093629 18154502

[45] Population Research Laboratory. Queensland Social Survey. Central Queensland University, Australia: Institute for Health and Social Science Research; 2012.

[46] Australian Bureau of Statistics. Census of Population and Housing: Census Tables [Internet]. 2011 [cited 23 Jul 2014]. Available: http://www.abs.gov.au/census

[47] Quandt S, Verhoef M, Arcury T, Lewith G, Steinsbekk A, Kristoffersen A, et al Development of an international questionnaire to measure use of complementary and alternative medicine (I-CAM-Q). J Altern Complement Med. 2009;15: 331–339. 10.1089/acm.2008.0521 19388855 PMC3189003

[48] MacLennan AH, Wilson DH, Taylor AW. The Escalating Cost and Prevalence of Alternative Medicine. Prev Med. 2002;35: 166–173. 10.1006/pmed.2002.1057 12200102

[49] Barnes PM, Powell-Griner E, McFann K, Nahin RL. Complementary and alternative medicine use among adults: United States, 2002. Semin Integr Med. 2004;2: 54–71. 10.1016/j.sigm.2004.07.003 15188733

[50] Boon H, Stewart M, Kennard MA, Gray R, Sawka C, Brown JB, et al Use of Complementary/Alternative Medicine by Breast Cancer Survivors in Ontario: Prevalence and Perceptions. J Clin Oncol. 2000;18: 2515–2521. 10893281 10.1200/JCO.2000.18.13.2515

[51] Wallston KA, Wallston BS, DeVellis R. Development of the Multidimensional Health Locus of Control (MHLC) Scales. Health Educ Behav. 1978;6: 160–170. 10.1177/109019817800600107 689890

[52] Ellison CG, Bradshaw M, Roberts CA. Spiritual and religious identities predict the use of complementary and alternative medicine among US adults. Prev Med. 2012;54: 9–12. 10.1016/j.ypmed.2011.08.029 21893085 PMC3770857

[53] Hildreth KD, Elman C. Alternative Worldviews and the Utilization of Conventional and Complementary Medicine. Sociol Inq. 2007;77: 76–103.

[54] Gosling SD, Rentfrow PJ, Swann WB Jr.. A very brief measure of the Big-Five personality domains. J Res Personal. 2003;37: 504–528. 10.1016/S0092-6566(03)00046-1

[55] Toplak ME, West RF, Stanovich KE. Assessing miserly information processing: An expansion of the Cognitive Reflection Test. Think Reason. 2014;20: 147–168. 10.1080/13546783.2013.844729

[56] Pennycook G, Cheyne JA, Seli P, Koehler DJ, Fugelsang JA. Analytic cognitive style predicts religious and paranormal belief. Cognition. 2012;123: 335–346. 10.1016/j.cognition.2012.03.003 22481051

[57] Koehler DJ, James G. Probability matching and strategy availability. Mem Cognit. 2010;38: 667–676. 10.3758/MC.38.6.667 20852231

[58] Australian Government Department of Health. National Immunisation Strategy for Australia 2013–2018 [Internet]. Commonwealth of Australia; 2013. Available: http://www.immunise.health.gov.au/internet/immunise/publishing.nsf/Content/immunisation-strategy-2013-18-cnt

[59] Browne M, Pennycook G, Goodwin B, McHenry M. Reflective minds and open hearts: Cognitive style and personality predict religiosity and spiritual thinking in a community sample. Eur J Soc Psychol. 2014;44: 736–742. 10.1002/ejsp.2059

[60] Shenhav A, Rand DG, Greene JD. Divine intuition: Cognitive style influences belief in God. J Exp Psychol Gen. 2012;141: 423–428. 10.1037/a0025391 21928924

[61] Swami V, Voracek M, Stieger S, Tran US, Furnham A. Analytic thinking reduces belief in conspiracy theories. Cognition. 2014;133: 572–585. 10.1016/j.cognition.2014.08.006 25217762

[62] Ajzen I, Timko C. Correspondence Between Health Attitudes and Behavior. Basic Appl Soc Psychol. 1986;7: 259–276. 10.1207/s15324834basp0704_2

